# Methicillin-Resistant and Biofilm-Producing *Staphylococcus aureus* in Nasal Carriage among Health Care Workers and Medical Students

**DOI:** 10.1155/2023/8424486

**Published:** 2023-01-04

**Authors:** Bhuvan Saud, Gita Khatri, Neetu Amatya, Govinda Paudel, Vikram Shrestha

**Affiliations:** Department of Medical Laboratory Technology, Janamaitri Foundation Institute of Health Sciences, GPO Box 8322, Hattiban, Lalitpur, Nepal

## Abstract

Antimicrobial resistance (AMR) is a global threat. It has been portrayed as a slow tsunami. Multidrug resistance and extensive drug resistance exacerbate the already-existing AMR problem. The aim of the study was to access the colonization of methicillin-resistant and biofilm-producing* Staphylococcus aureus* among healthcare workers (HCWs) and medical students (MSs). A cross-sectional study was designed. A total of 352 participants (176 were HCWs and 176 were MSs) were enrolled from different hospitals and medical colleges in Kathmandu, Nepal. Nasal cavity swab samples were collected and inoculated on Mannitol salt agar at standard in-vitro environmental conditions. Isolates were identified based on colony characteristics, staining properties, and biochemical tests. Identified isolates were tested for antibiotic susceptibility and biofilm production. Out of 352 participants, 65.3% were *S. aureus* carriers; among the carriers, 52.2% were HCWs and 47.8% were MSs. Of the total isolates, 47.4% isolates were methicillin-resistant* S. aureus* (MRSA) and 73.9% isolates were multidrug-resistant (MDR). Among MDR isolates, out of 109 MRSA isolates, 86.2% were MDR and out of 121 MSSA isolates, 62.8% were MDR where isolates were mainly resistant to erythromycin. In addition, 68.7% isolates were biofilm-forming; the results were similar in both MRSA and MSSA. Variables such as profession and educational level showed statistical significance (*p* < 0.05) with MRSA, MSSA, and biofilm producers. In conclusion, asymptomatic colonization of healthcare workers by drug-resistant *S. aureus* is increasing at alarming rates. This reflects the lack of proper hygiene practice as well as improper disinfection of workplace of study population.

## 1. Introduction

*Staphylococcus aureus* is a microbiota of human body; however, it can cause mild to fatal infections. Globally, around 30.0% and 50.0% of people carry *S. aureus* in their nasal cavities permanently [1, 2]. In the early 1960s, methicillin-resistant *S. aureus* (MRSA) was first reported in England [3], which is now ubiquitous all around the world. MRSA is a group of Gram-positive bacteria that genetically differ from other strains of *S. aureus* that cause an infection which does not respond to common antibiotics, including methicillin, amoxicillin, and penicillin [4]. Methicillin resistance is mediated by acquiring mecA gene on *S. aureus* chromosomal DNA which confers resistance to *β*-lactam antibiotics. Worldwide, the prevalence of MRSA varies from 13.0% to 74.0% [5], while in the South-East Asian and Western Pacific areas, prevalence varied from 2.0% to 69.0% [6]. In Nepal, MRSA distribution ranges from 46.0% to 57.8% [7–10]. *S. aureus* has an ability to form biofilm, that acts in defense mechanism, enhances virulence properties, and is also associated with several infections such as bacteremia, sepsis, and endocarditis [11]. More than 14 adhesion genes are involved in bacterial cellular aggregation and accumulation [12]. It was reported that biofilm-forming* Staphylococci* can resist antibiotic concentration 10–10,000 times higher than the free-floating ones [13]. Also, studies in Nepal elucidated that prevalence of biofilm producing *S. aureus* range from 69.8% to 100.0% [8, 14].

Nasal cavity is one of the ecological niches for *S. aureus*. Host characteristics and environmental factors can predispose to colonization of bacteria, even though majority of hosts remain asymptomatic carriers. Hospital environment, gowns, patients' care items, etc., contribute prominently to the pathogen transmission in healthcare setting. A study from England concluded that 58.0% healthcare workers (Healthcare worker: According to The Public Health Service Act 2018, a healthcare worker is defined as anyone who registered in the concerned council as a healthcare worker under the prevailing law of Nepal) (HCWs) detected *S. aureus* carrier in nasal cavity [15]. In a hospital setting, HCWs and medical students (Medical student: a medical student is a person who is enrolled in an educational institution to pursue a medical education degree). MSs act as an asymptomatic carrier of the pathogen and transmit infection in community and hospital settings. Regular screening of methicillin-resistant and biofilm-producing *S. aureus* in HCWs and MSs could minimize the potential outbreak of the pathogen. Hitherto, in case of Nepal, limited studies have been conducted to determine the prevalence of methicillin-resistant and biofilm-producing *S. aureus* among HCWs and MSs. Thus, this study aimed to investigate the asymptomatic colonization of methicillin-resistant and biofilm-producing *S. aureus* among HCWs and MSs in Kathmandu, Nepal.

## 2. Materials and Methods

### 2.1. Study Design, Study Area, and Sampling

A cross-sectional study was conducted between January, 2022, and June, 2022, in Kathmandu. Samples were recruited by a stratified-random sampling technique. A total of 352 volunteer healthcare personnel (176 HCWs and 176 MSs) were enrolled. The sample size was calculated using the formula *n* = *Z*2*α*/2*pqN*/*e*2 (*N* − 1) + *Z*2*α*/2*pq*, where *α*/2 = level of significance at 95% confidence interval (or 5% level of significance) and *Zα*/2 = 1.96. *p* = proportion/prevalence of *S. aureus* was 35.3% [16], thus *p* = 0.5, *q* = 0.5, *N* = total population size of the study area, and *e* = maximum allowance error which is assumed at 0.05. Nasal swab samples were collected from participants and a face-to-face interview was performed for sociodemographic characterization. Samples were processed and examined in the Department of Medical Laboratory Technology, Janamaitri Foundation Institute of Health Sciences, Lalitpur, Nepal.

### 2.2. Bacterial Isolation and Characterization

Swab samples were inoculated onto Mannitol salt agar and Nutrient Agar incubated at 37°C for 24 hours. *S. aureus* colonies were identified based on their Gram's staining properties, colony characteristics, and biochemical tests (catalase, coagulase, oxidase, and deoxyribonuclease tests).

### 2.3. Antimicrobial Susceptibility Test

Antimicrobial susceptibility was performed in Muller–Hinton agar via the modified Kirby-Bauer disc diffusion method adopting Clinical and Laboratory Standards Institute guidelines [17]. Commercially available following antibiotic discs were used: amoxicillin (10 *μ*g), amikacin (30 *μ*g), ciprofloxacin (5 *μ*g), cotrimoxazole (1.25 *μ*g), erythromycin (15 *μ*g), and tetracycline (30 *μ*g). Cefoxitin (30 *μ*g) inhibitory zone diameter ≤ 21 mm around the disc was interpreted as an MRSA strain. *S. aureus* ATCC 25923 control strain was used for quality control.

### 2.4. Screening of Biofilm Production

The tissue culture plate method was employed for detection of biofilm-producing* S. aureus* and a quantitative test was performed as published elsewhere [14, 18].

### 2.5. Statistical Analysis

Data were entered and analyzed in Statistical Package for the Social Sciences (IBM Corp., USA) version 21. Chi-square test was performed for categorical data and a *p* value less than 0.05 was considered significant.

## 3. Results

### 3.1. Sociodemographic Characteristics, Prevalence of *S. aureus*, and Association with MRSA and MSSA

An equal number of HCWs and MSs (176 in each) were engaged, where females (82.1%) were higher than the male (17.9%) participants and mean age was 27.6 years. The detailed demographic distributions and clinical traits are presented in Table 1. Out of 352 participants, 230 (65.3%) were positive for *S. aureus* carriage. In HCWs and MSs, *S. aureus* was isolated in 120 (52.2%) and 110 (47.8%) participants, respectively. The rate of nasal carriage was higher in female (83.0%) than the male (16.9%) participants. Out of 230 bacterial isolates, 109 (47.4%) were found to be MRSA while MSSA were 121 (52.6%). Furthermore, 38 (34.9%) MRSA and 38 (31.4%) MSSA isolates exhibited strong biofilm-forming potency whereas 17 (15.6%) of MRSA and 20 (16.5%) of MSSA showed a moderate level of biofilm production. All biofilm producers MRSA and MSSA were highly resistant to erythromycin whilst strong biofilm-forming MRSA was also equally resistant to amoxicillin. However, there was no significant relation with the level of biofilm producers MRSA and MSSA with antibiotic resistance.

### 3.2. Multidrug Resistance Pattern

Multidrug resistance (MDR) is defined as acquired nonsusceptibility to at least one agent in three or more antimicrobial categories [19]. A total of 230 *S. aureus* were isolated in which 170 (73.9%) isolates were MDR. Out of which, the maximum number of bacteria was resistant to two and three classes of antibiotics (*n* = 65 in both cases) while resistance to four drugs was seen in 24 isolates, resistance to five was seen in 14 isolates, and resistance to six was seen in 2 isolates. Likewise, among 121 MSSA isolated, 76 (62.8%) were MDR where 26 showed resistance to three different classes of antibiotics, 6 showed resistance to four classes of antibiotics, and 4 showed resistance to five drugs in varying patterns. Additionally, it was found that out of 109 MRSA, 94 (86.2%) were MDR of which 39 isolates displayed resistance to three classes of antibiotics, 18 displayed resistance to four classes of antibiotics, and 10 displayed resistance to five classes drugs and 2 to six classes.

### 3.3. Antibiotic Resistance Pattern in MRSA and MSSA

Most of the isolates were resistant to erythromycin in both MRSA (82.5%) and MSSA (68.6%). Meanwhile, second-highest resistance was seen for amoxicillin in MRSA (73.4%) isolates and cotrimoxazole in MSSA (45.5%) as shown in Table 2. However, the correlation was statistically insignificant (*p* value > 0.05).

### 3.4. Biofilm-Producing*S. aureus*

Out of 230 isolates, 72 (31.3%) were nonbiofilm-producing*S. aureus* and 158 (68.7%) were biofilm-producing*S. aureus*. Out of total biofilm producers, MSSA (50.6%) produced biofilms almost equally to their MRSA (49.4%) counterpart. An equal number of isolates were strong biofilm producers in both MSSA and MRSA. In the moderate biofilm producer category, the number of MSSA isolates was higher than MRSA and in the weak biofilm producer group, MRSA isolates were higher than MSSA. There was, however, no significant relation (*p* = 0.794) observed. Details are shown in Table 3.

## 4. Discussion

The nasal carriage rate of *S. aureus* among the participants was 65.3%, which is higher than that reported in studies from elsewhere in Nepal; 14.7%–15.7% [20, 21]. Similarly, the prevalence of MRSA (47.4%) was higher than that in studies published from different settings of Nepal; 2.3%–41.3% [16, 22–25]. A meta-analysis study, between 2008 and 2020 in Nepal, noted that prevalence of MRSA ranged from 14.6% to 70.6% [26]. However, other studies from Nepal and Kenya have found a higher MRSA prevalence rate of 57.0% [11] and 53.4% [27] than ours. In this study, the high prevalence of MRSA could be because participants were healthcare personnel. Also, studies have found that Methicillin-resistantCoagulase Negative Staphylococci (MR-CoNS) acts a potential reservoir of mecA gene that facilitates MRSA colonization in human via horizontal crosspropagation of resistant genes when co-colonized resulting high prepondance of MRSA in nasal mucosa [28, 29]. In addition, sufficient practices related to infection control and preventive measures, extensive and excessive antibiotic usage, and low socioeconomic status increase the chance of MRSA carriage.

In this study, 73.9% isolates were MDR and mainly resistant to erythromycin followed by amoxicillin and cotrimoxazole. Out of 109 MRSA isolates, 94 (86.2%) were MDR. A similar study has noted that all the MRSA isolates were MDR [23]. Furthermore, biofilm producer *S. aureus* is two folds higher in number than the nonbiofilm producers. However, biofilm producers were nearly equal in numbers in both MRSA and MSSA. On the contrary, a report from Poland has found that MRSA isolates have a higher ability to produce biofilms than MSSA [30]. Biofilms provide a protective barrier against the host defense, disinfectants, and antibiotics. It has been noted that incidence of methicillin resistance was nearly two folds higher in biofilm producers than the nonproducers [20]. Several other human pathogenic bacteria such as *S. epidermidis*, *Enterococcus faecalis*, *Viridans streptococci*, *Escherichia coli*, *Proteus mirabilis*, *Klebsiella pneumoniae*, and *Pseudomonas aeruginosa* produce biofilms [31]. Biofilm-producing bacteria are accountable for increased pathogenicity, long hospital stay, treatment failure with current antibiotic drugs, economic crisis [32], and may even contribute cancer development [33]. In our study, biofilm-producing*S. aureus* was mainly resistance to erythromycin and amoxicillin. Other studies noted that *S. aureus* isolates were mostly resistant to penicillin and erythromycin [34].

HCWs and MSs frequently encounter potentially lethal microorganism from patients and hospital environments. Therefore, they may transmit the disease to another person in hospital and community. A study revealed that the prevalence of MRSA is higher mainly in doctors and nurses [16]. In our study, there was a significant relation to the education level and profession with MRSA colonization. A similar study evidenced that the education level was significantly associated with the colonization of MRSA [35]. A study from Ethiopia reported that nasal carriage of MRSA was significantly associated with exposure time in hospital, sharing of clothing and sports equipment, and nose picking habit [36]. However, no significant relation to risk factors such as gender, age, occupation, experience, nasal irritation, and history of upper respiratory tract infection with MRSA colonization was recorded from Nepal [16].

Colonization of methicillin-resistant and biofilm-producing* S. aureus* could cause fatal infection in immunosuppressive clinical conditions. Asymptomatic colonization of the bacteria in HCWs and MSs could transmit the infection to hospitalized patients. *S. aureus* easily spreads through touching with contaminated hands. Periodic screening, decolonization, and adequate awareness among health professionals about the nasal carriages could minimize the risk of spread. Screening is also important in area where there is lack of sophisticated healthcare system, poor hygiene practice, and inadequate awareness.

Some constraints of this study are that the targeted population were all health professional which might have caused selection bias for high prevalence of MRSA. Also, this study has not examined co-colonization of MR-CoNS and MRSA as some studies have suggested that MR-CoNS are the repository for mecA gene and enhances MRSA colonization [28, 29]. Furthermore, the identification of MRSA and biofilm detection were performed using conventional methods due to the lack of molecular laboratory.

## 5. Conclusion

Prevalence of methicillin-resistant, biofilm-producing, and multidrug-resistant *S. aureus* high among HCWs and MSs. Both the methicillin-resistant and biofilm producers are resistant to multiple antibiotics than nonmethicillin-resistant and nonbiofilm producers. Hygiene among healthcare professionals should be highly emphasized in infection control and prevention strategies. The high prevalence of MRSA underscores the necessity for an effective surveillance system in Nepal.

## Figures and Tables

**Table 1:** Comparison of sociodemographic variables with MRSA and MSSA colonization among participants.

Variables	Distribution (*N* = 352, %)	*S. aureus* (*N* = 230, %)	MRSA (*N* = 109, %)	MSSA (*N* = 121, %)	*p* value
*Gender*					
Male	63 (17.9)	39 (16.9)	18 (16.5)	21 (17.4)	0.865
Female	289 (82.1)	191 (83.0)	91 (83.5)	100 (82.6)	
*Age group (years)*					
20–29	278 (79.0)	178 (77.4)	83 (76.1)	95 (78.5)	0.331
30–39	46 (13.1)	34 (14.8)	16 (14.7)	18 (14.9)	
40–49	22 (6.3)	14 (6.1)	6 (5.5)	8 (6.6)	
50–59	5 (1.4)	3 (1.3)	3 (2.8)	0 (0.0)	
60–69	1 (0.3)	1 (0.4)	1 (0.9)	0 (0.0)	
*Category*					
HCW	176 (50.0)	120 (52.2)	56 (51.4)	64 (52.9)	0.818
MS	176 (50.0)	110 (47.8)	53 (48.6)	57 (47.1)	
*Profession*					
Doctor	20 (5.7)	15 (6.5)	6 (5.5)	9 (7.4)	0.002^∗^
Nurse	67 (19.0)	49 (21.3)	33 (30.3)	16 (13.2)	
Lab personnel	14 (4.0)	9 (3.9)	1 (0.9)	8 (6.6)	
Pharmacist	8 (2.3)	4 (1.7)	3 (2.8)	1 (0.8)	
Others	67 (19.0)	42 (18.3)	13 (11.9)	29 (24.0)	
Students	176 (50.0)	111 (48.3)	53 (48.6)	58 (47.9)	
*Education level*					
Literate	30 (8.5)	22 (9.6)	5 (4.6)	17 (14.0)	0.005^∗^
Diploma	20 (5.7)	12 (5.2)	3 (2.8)	9 (7.4)	
Graduate	271 (77.0)	175 (76.1)	86 (78.9)	89 (73.6)	
≥Master	31 (8.8)	21 (9.1)	15 (13.8)	6 (5.0)	
*Duration of work (years)*					
<1	182 (51.7)	113 (49.1)	53 (48.6)	60 (49.6)	0.519
1–10	144 (40.9)	98 (42.6)	45 (41.3)	53 (43.8)	
11–20	20 (5.7)	14 (6.1)	7 (6.4)	7 (5.8)	
21–30	6 (1.7)	5 (2.2)	4 (3.7)	1 (0.8)	
*Duty hours per week*					
48	176 (50.0)	120 (52.2)	56 (51.4)	64 (52.9)	0.818
6	176 (50.0)	110 (47.8)	53 (48.6)	57 (47.1)	
*Upper respiratory tract symptoms*					
Present	17 (4.8)	7 (3.0)	3 (2.8)	4 (3.3)	0.807
Absent	335 (95.2)	223 (97.0)	106 (97.2)	117 (96.7)	
*Nasal irritation*					
Present	32 (9.1)	22 (9.6)	13 (11.9)	9 (7.4)	0.248
Absent	320 (90.9)	208 (90.4)	96 (88.1)	112 (92.6)	
*Underlying disease*					
Present	9 (2.6)	9 (3.9)	6 (5.5)	3 (2.5)	0.237
Absent	343 (97.4)	221 (96.1)	103 (94.5)	118 (97.5)	
*Family respiratory disease*					
Present	27 (7.7)	18 (7.8)	6 (5.5)	12 (9.9)	0.213
Absent	325 (92.3)	212 (92.2)	103 (94.5)	109 (90.1)	
*Nasal hygiene*					
Present	124 (35.2)	79 (34.3)	39 (35.8)	40 (33.1)	0.664
Absent	228 (64.8)	151 (65.7)	70 (64.2)	81 (66.9)	

^∗^Statistically significant.

**Table 2:** Antibiotic resistance between MRSA and MSSA.

Antibiotics	*S. aureus* (*N* = 230)		*p* value
	MRSA (*N* = 109)	MSSA (*N* = 121)	
	No. of resistant isolates (%)	No. of resistant isolates (%)	
Erythromycin	90 (82.6)	83 (68.6)	0.391
Tetracyclin	19 (17.4)	15 (12.4)	
Amikacin	27 (24.8)	14 (11.6)	
Amoxicillin	80 (73.4)	51 (42.2)	
Cotrimoxazole	68 (62.4)	55 (45.5)	
Ciprofloxacin	32 (29.4)	16 (13.2)	
MDR	94 (86.2)	76 (62.8)	

**Table 3:** Distribution of biofilm forming MSSA and MRSA.

Type of biofilm producer	*S. aureus*		*p* value
	MRSA (*N*, %)	MSSA (*N*, %)	
Biofilm nonproducer	31 (28.4)	41 (33.9)	0.794
Weak biofilm producer	23 (21.1)	22 (18.2)	
Moderate biofilm producer	17 (15.6)	20 (16.5)	
Strong biofilm producer	38 (34.9)	38 (31.4)	

## Data Availability

The data used to support the finding of this study are available from the corresponding author upon request to authors.

## Abbreviation

*S. aureus*: *Staphylococcus aureus*
HCWs: Healthcare workers
MSs: Medical students
MDR: Multidrug-resistant
MRSA: Methicillin-resistant*S. aureus*
MSSA: Methicillin-sensitive*S. aureus*
MR-CoNS: Methicillin-resistant coagulase negative *S. aureus*
*N*: Number.
